# Supplementary material for: In silico comparative genomics of SARS-CoV-2 to determine the source and diversity of the pathogen in Bangladesh
Source: PLoS One. 2021 Jan 20;16(1):e0245584. doi: 10.1371/journal.pone.0245584 (PMC7817022; doi:10.1371/journal.pone.0245584)
Supplement: S1 Table — (DOCX) [file pone.0245584.s001.docx]

**S1 TABLE: NUMBER OF SEQUENCES FORM DIFFERENT COUNTRIES**

| **Country** | **Number of Sequences** |
| --- | --- |
| African region | 17 |
| Australia | 27 |
| Bangladesh | 65 |
| Brazil | 15 |
| Canada | 19 |
| China | 31 |
| France | 20 |
| Germany | 20 |
| India | 18 |
| Iran | 5 |
| Italy | 30 |
| Mexico | 8 |
| Nepal | 1 |
| Pakistan | 3 |
| Russia | 14 |
| Saudi Arabia | 19 |
| Spain | 15 |
| Sri Lanka | 6 |
| Sweden | 5 |
| Turkey | 10 |
| United Kingdom | 31 |
| USA | 55 |
